# Study on the Suppression Mechanism of Vibration and Noise in Converter Transformers Under Multi-Physical Field Coupling Using Different Filtering Strategies

**DOI:** 10.3390/s26185903

**Published:** 2026-09-17

**Authors:** Yuzhuo Wang, Liwei Xie, Sheng Xiang

**Affiliations:** School of Electrical and Information Engineering, Changsha University of Science and Technology, Changsha 410114, China; wangoddd@163.com (Y.W.); sxiang@csust.edu.cn (S.X.)

**Keywords:** converter transformer, harmonic filtering, electromagnetic force, vibration and noise, acoustic radiation, multiphysical field coupling

## Abstract

Electromagnetic vibration and noise issues of converter transformers under harmonic operating conditions have become increasingly prominent. To enable a systematic comparison of different filtering strategies, this paper establishes a multiphysical field coupling model incorporating electromagnetic, structural, and acoustic fields, and systematically investigates four filtering configurations: grid-side LC filtering, valve-side LC filtering, inductive filtering, and valve-side capacitive filtering through theoretical analysis, finite-element simulation, and experimental verification on a 12-pulse laboratory HVDC prototype platform. The simulation results show that the maximum vibration acceleration decreases from 3.06 m/s^2^ under the no-filter condition to 1.02 m/s^2^ under valve-side capacitive filtering. Experimental measurements further confirm the suppression effect, with the vibration acceleration at Point 8 decreasing from 2.28 m/s^2^ to 0.42 m/s^2^, corresponding to a reduction of approximately 82%, while the average A-weighted sound pressure level decreases from 66.1 dB to 58.1 dB. These results indicate that vibration and noise suppression cannot be evaluated solely by total harmonic distortion; the attenuation and distribution of individual harmonic components should also be considered. The findings provide a theoretical and engineering basis for the vibration and noise reduction design of converter transformers.

## 1. Introduction

High-voltage direct current (HVDC) transmission technology, leveraging its advantages in long-distance, large-capacity power transmission and renewable energy integration, has become a core component of modern power systems [1]. However, the power electronic devices used for alternating-current/direct-current (AC/DC) conversion exhibit nonlinear switching characteristics, which inevitably generate characteristic harmonic currents that are injected into the AC system [2,3]. When these harmonic currents flow through the converter transformer, they not only increase electrical losses and temperature rise, but also induce intense electromagnetic vibrations and audible noise [4,5,6]. Specifically, the interaction between harmonic currents and the main magnetic flux intensifies the magnetostrictive effect of the core on the one hand, and enhances the fluctuation in electromagnetic forces in the windings on the other, thereby significantly increasing the overall vibration level of the transformer. Such severe vibration not only causes noise pollution, affecting the surrounding environment, but may also pose a potential threat to the long-term mechanical reliability of the transformer [7,8,9].

To suppress harmonics and the resulting vibration noise, filtering technology is commonly employed in engineering practice. However, different filtering schemes exhibit significant differences in their design intentions, working principles, and suppression effects on transformer body vibration. As a traditional solution, LC passive filters have been extensively researched and applied, with existing literature primarily focusing on their parameter design, resonance risks, and improvements to grid-side power quality [10,11,12]. Reference [13] proposes a high-power LC hybrid active power filter (HAPF), which can meet the requirements of filtering and dynamic reactive power compensation. Reference [14] proposes a parameter optimization method for AC filters (ACFs), which takes into account both technical performance and economic cost. The comparison results show that the optimized ACF effectively improves and ensures performance in reactive power compensation and filtering, while also reducing costs. However, the LC filter is located on the grid side of the transformer, with its primary objective being to prevent harmonic currents from flowing into the power grid. It can only provide indirect suppression and has inherent limitations. Currently, few studies have quantitatively evaluated its actual suppression effectiveness on the vibration and noise of the converter transformer itself.

Inductive filtering technology, on the other hand, offers an innovative “diversion” approach. By incorporating additional harmonic compensation windings inside the transformer and connecting external tuning branches, it provides a low-impedance path for harmonic currents, generating a magnetic field that cancels the harmonic magnetomotive force from the valve-side windings [15,16,17]. Reference [18] conducted an electromagnetic vibration analysis of the windings of an inductive filtering converter transformer. The winding currents were calculated using multi-winding transformer theory, and then the Lagrangian equations were applied to compute the radial and axial electromagnetic forces on the three windings. A three-dimensional finite element model of the inductive filtering converter transformer was established. Transient dynamic (time-domain) simulation results show that the inductive filtering converter transformer and its corresponding filters can suppress electromagnetic vibration of the windings. However, the paper only analyzed winding vibration without analyzing noise, and no systematic comparison with other schemes was performed under the same benchmark.

The valve-side capacitive filtering scheme involves connecting capacitor banks directly to the valve side of the converter transformer, thereby providing a local low-impedance shunt path for harmonic currents generated by the converter. This configuration reduces the harmonic current components entering the transformer windings and therefore represents a source-side harmonic suppression approach. Reference [19] analyzes the role of fixed shunt capacitors in improving commutation and harmonic filtering performance and provides a basis for parameter selection. Real-time digital simulation results verify the effectiveness of the proposed scheme in improving commutation performance, system stability, and harmonic filtering, while also evaluating the electrical stresses on the fixed shunt capacitors. References [20,21] further investigate reactive-power and AC-voltage control on the inverter side of a line-commutated-converter-based HVDC (LCC-HVDC) system equipped with controllable capacitors. By enabling operation at negative extinction angles, the system achieves an extended reactive-power control range, including reactive-power output. However, these studies mainly focus on commutation performance, system stability, harmonic filtering, and reactive-power/voltage control, without considering the vibration and noise response of the converter transformer. Reference [22] proposes a capacitor-filter-based HVDC converter to reduce the vibration and noise of the converter transformer. Experimental results show that, compared with conventional LCC operation, the capacitor-filter configuration substantially reduces transformer vibration and noise. However, although quantitative reductions in harmonic distortion and noise were reported, the comparison was limited to conventional LCC operation and capacitive-filter operation. A systematic comparison of capacitive filtering with other representative filtering strategies under identical experimental conditions therefore remains lacking. Beyond electrical filtering approaches, active structural vibration-control methods, such as adaptive-switching fuzzy control, have also demonstrated effective vibration suppression under uncertain and time-varying conditions [23]. The present study, however, focuses specifically on converter transformers and investigates vibration and noise suppression from the perspective of filtering configuration and harmonic excitation.

In summary, the current research exhibits the following characteristics and deficiencies: First, investigations into the vibration and noise mechanisms of transformers under harmonic excitation mostly remain at the simulation and theoretical levels, and are insufficiently integrated with experimental validation of specific filtering and suppression technologies. Recent studies have also advanced multi-field and electromechanical coupling models for transformer vibration and vibroacoustic analysis [24,25,26]. Second, the performance evaluation of filtering technologies is primarily based on electrical indicators such as total harmonic distortion (THD), and rarely extends to mechanical vibration and acoustic noise, which directly affect equipment service life and the surrounding environment. Third, there is a lack of systematic comparative experiments; systematic quantitative comparisons and mechanistic analyses of the aforementioned filtering schemes under identical experimental conditions remain limited, particularly those using the vibration acceleration and sound pressure level of the converter transformer itself as direct observation targets.

The methodological contribution of this study is to integrate established electromagnetic, structural, and acoustic formulations into a system-level analytical framework linking filtering configuration, harmonic-spectrum variation, structural vibration, and acoustic response. By constructing an HVDC laboratory prototype platform to replicate the harmonic excitation environment under representative operating conditions, the vibration and noise characteristics of the converter transformer under four filtering configurations, namely grid-side LC filtering, valve-side LC filtering, inductive filtering, and valve-side capacitive filtering, are systematically studied and compared. The experiments quantitatively measure the vibration acceleration and sound pressure level under different filtering configurations, evaluate the actual suppression effectiveness of each scheme on vibration and noise, and conduct an in-depth analysis of the underlying mechanisms in conjunction with electrical measurement results. The research findings aim to provide direct experimental evidence and theoretical support for the selection and parameter optimization of filtering schemes for vibration and noise mitigation in converter transformers in engineering practice.

The remainder of this paper is organized as follows. Section 2 establishes the analytical relationship among harmonic excitation, electromagnetic force, structural vibration, and acoustic radiation, and analyzes the suppression mechanisms of different filtering strategies. Section 3 introduces the 12-pulse HVDC laboratory prototype platform, measurement system, and sensor arrangement. Section 4 presents and discusses the electrical, vibration, and acoustic measurement results under different filtering conditions. Finally, Section 5 summarizes the main conclusions of this study.

## 2. Mechanisms and Suppression Methods of Vibration and Noise in Converter Transformers Under Harmonic Excitation

### 2.1. Mechanism of Vibration and Noise Generation Under Harmonic Current Excitation

#### 2.1.1. Harmonic Excitation of Converter Transformer

The instantaneous current of the converter transformer contains characteristic harmonics and can be expressed as the following time-varying function [27]:

(1) $$i(t)=\sqrt{2}\left[I_{1}\sin(\omega_{1}t+\varphi_{1})+\sum_{h\in H}I_{h}\sin(\omega_{h}t+\varphi_{h})\right]$$

where *h* denotes the characteristic harmonic order, and *H* denotes the set of characteristic harmonic orders generated by the 12-pulse converter, expressed as *H* = {12k ± 1 ∣k ∈ Z^+^}, where k is a positive integer. The corresponding angular frequency is ω*_h_* = *h*_1_ω_1_, ω_1_ = 2π*f*_1_, *f*_1_ = 50 Hz.

#### 2.1.2. Derivation of the Excitation Force Density Due to Magnetostriction in the Iron Core

Magnetostriction is a physical effect in which ferromagnetic materials undergo elastic deformation under the influence of a magnetic field. For silicon steel sheets, the magnetostrictive strain *λ*(*t*) along the rolling direction and the magnetic flux density *B*(*t*) satisfy a complex nonlinear relationship, which can generally be approximated by a power series [28]:

(2) $$\lambda (t)=\sum_{n=1}^{\infty }k_{n}B{\left(t\right)}^{n}$$

where *k_n_* are the material-dependent magnetostriction coefficients. In engineering analysis, taking up to the quadratic term is usually sufficient to capture the main characteristics:

(3) $$\lambda (t)\approx k_{1}B(t)+k_{2}B{\left(t\right)}^{2}$$

The first term *k*_1_*B*(*t*) is the linear term (magnetoelastic effect), and the second term *k*_2_*B*(*t*) is the nonlinear term, which constitutes the main source term generating vibration.

Assume that the magnetic flux density along the magnetization direction in the iron core is:

(4) $$B(t)=B_{1}\sin(\omega_{1}t)+\sum_{h\in H}B_{h}\sin(\omega_{h}t+\delta_{h})$$

Substituting it into the quadratic term:

(5) $$\begin{array}{l} B{\left(t\right)}^{2}={\left[B_{1}sin(\omega_{1}t)+\sum_{h}B_{h}\sin(\omega_{h}t+\delta_{h})\right]}^{2}\\ \;=\frac{B_{1}^{2}}{2}\left[1-\cos(2\omega_{1}t)\right]+\sum_{h}\frac{B_{h}^{2}}{2}\left[1-\cos(2\omega_{h}t+2\delta_{h})\right]\\ \;+\;\sum_{h}B_{1}B_{h}\left[\cos((\omega_{h}-\omega_{1})t+\delta_{h})-\cos((\omega_{h}+\omega_{1})t+\delta_{h})\right]\\ \;+\;\sum_{h\neq l}B_{h}B_{l}\left[\cos((\omega_{h}-\omega_{1})t+\delta_{h}-\delta_{l})-\cos((\omega_{h}+\omega_{1})t+\delta_{h}+\delta_{l})\right] \end{array}$$

It can be seen that the excitation force density *f*(*t*) generated by magnetostriction (which is proportional to the second time derivative of *λ*(*t*)) will contain the following frequency components:

(1) Fundamental frequency multiples: 2*f*_1_ and 2*f_h_*. For example, when *f*_1_ is 50 Hz and *f_h_* is 550 Hz, 2*f*_1_ yields 100 Hz and 2*f_h_* yields 1100 Hz.
(2) Sum and difference frequencies: *f_h_* ± *f*_1_. For example, the interaction between 550 Hz and 50 Hz produces frequency components at 500 Hz and 600 Hz.
(3) Sum and difference frequencies among harmonics: *f_h_* ± *f*_l_. For example, the interaction between 550 Hz and 650 Hz produces a sum-frequency component at 1200 Hz and a difference-frequency component at 100 Hz, where the latter is obtained by taking the absolute value of −100 Hz.

The instantaneous excitation force density of the iron core can be modeled as:

(6) $$f(r,t)=-\nabla \cdot \sigma_{ms}(r,t)$$

where *σ_ms_* is the magnetostrictive stress tensor. Under the approximation of uniform isotropy, *σ_ms,ij_* ∝ *B_i_B_j_*. This force density serves as a source term in the structural dynamics equation of the iron core.

#### 2.1.3. Derivation of the Lorentz Force Density in the Windings

The interaction between the current density **J**(**r**,*t*) in the windings and the leakage flux density **B***_σ_*(**r**,*t*) generates the Lorentz volume force density:

(7) $$f_{wind}(r,t)=J(r,t)\times B_{\sigma }(r,t)$$

The leakage flux density **B***_σ_* itself is established by the total load current *i*(*t*) under the linear approximation, and its spatial distribution function **b***_σ_*(**r**) is proportional to the instantaneous value of the current:

(8) $$B_{\sigma }(r,t)=b_{\sigma }(r)\cdot i(t)$$

For windings with circular conductors, under axial (z-direction) symmetry conditions, the radial leakage flux density **B***_σ_*_,r_ dominates. The current density in the windings can be approximated as uniform (for low-frequency harmonics):

(9) $$J_{\theta }(r,t)\approx \frac{Ni(t)}{A}$$

where *N* is the number of turns, and *A* is the cross-sectional area of a single turn. Then the radial electromagnetic force density is:

(10) $$f_{r}(r,t)=J_{\theta }(t)\times B_{\sigma ,z}(r,t)\propto i(t)\cdot B_{\sigma ,z}(r,t)\propto i{\left(t\right)}^{2}$$

Therefore, the Lorentz force density on the windings is also proportional to the square of the current in the time domain. Expanding it:

(11) $$\begin{array}{l} i{\left(t\right)}^{2}=I_{1}^{2}\sin^{2}(\omega_{1}t)+\sum_{h}I_{h}^{2}\sin^{2}(\omega_{h}t)+2\sum_{h}I_{1}I_{h}\sin(\omega_{1}t)\sin(\omega_{h}t)\\ \;\;\;+2\sum_{h\neq l}I_{h}I_{l}\sin(\omega_{h}t)\sin(\omega_{l}t) \end{array}$$

Using trigonometric identities, force components at various frequencies are likewise generated, including a DC component, 2*f*_1_, 2*f_h_*, *f_h_* ± *f*_1_, *f_h_* ± *f*_l_ and others.

Under harmonic operating conditions, the transformer current can be expressed as the superposition of the fundamental wave and multiple harmonics, generating magnetostrictive forces in the core and Lorentz forces in the windings, respectively. Both types of forces are proportional to the square of the current, thus producing frequency-doubled components as well as sum- and difference-frequency components, which significantly broadens the vibration spectrum.

#### 2.1.4. Basic Noise Calculation

The electromagnetic force *f*(r,t) = *f*_core_ + *f*_wind_, acting as a distributed force source, excites vibrations in the solid structure of the transformer (core, windings, clamps, tank). The governing dynamic equation can be written as:

(12) $$\rho_{s}\frac{\partial^{2}u}{\partial t^{2}}-\nabla \cdot \sigma_{s}(u)=f(r,t)\;\mathrm{in}\;\Omega_{s}$$

where *ρ_s_* is the density of the structural material, *u* is the displacement field, *σ_s_* is the structural stress tensor (satisfying Hooke’s law *σ_s_* = C:ϵ, ϵ being the strain tensor), and Ω*_s_* is the structural domain.

The vibration displacement ***u****_w_*(*t*) of the tank wall serves as a boundary condition that drives the acoustic field in the surrounding fluid (air). Under the assumptions of acoustic far-field and small amplitude, the radiated sound pressure *p*(*x*,*t*) satisfies the Helmholtz equation:

(13) $$\nabla^{2}p-\frac{1}{c_{0}^{2}}\frac{\partial^{2}p}{\partial t^{2}}=0\;\;\mathrm{in}\;\Omega_{a}$$

where *c*_0_ is the speed of sound. On the structure–acoustic coupling interface Γ_sa_, the conditions of velocity continuity and force balance are satisfied:

(14) $$\frac{\partial p}{\partial n}=-\rho_{0}\frac{\partial^{2}u_{w}\cdot n}{\partial t^{2}}\;\;\mathrm{on}\;\Gamma_{sa}$$

where *ρ*_0_ is the air density, and ***n*** is the normal vector to the interface.

Finally, the sound pressure level *L_p_* at an observation point *x* is related to the spectral integral of the vibration velocity on the tank surface. For a single frequency component *ω_k_*, the radiated sound power is approximately proportional to the square of the vibration velocity amplitude and the radiation efficiency *σ*_rad_(*ω_k_*):

(15) $$W_{\mathrm{acoustic}\;}\left(\omega_{k}\right)\propto \sigma_{\mathrm{rad}}\left(\omega_{k}\right)\cdot {\left|v_{w}\left(\omega_{k}\right)\right|}^{2}$$

The radiation efficiency *σ*_rad_ is related to the vibration modes, frequency, and structural dimensions of the tank. When the excitation frequency approaches the natural frequency of the tank wall or internal structures, the vibration amplitude *v_w_* is sharply amplified due to resonance. Even if the excitation force is not large, this may still lead to significant noise peaks. Harmonic excitation increases the probability of matching the structural natural frequencies, which is another key mechanism by which harmonics exacerbate the noise problem.

### 2.2. Vibration and Noise Suppression Methods for Converter Transformers Using Different Filtering Approaches

**Figure 1:**
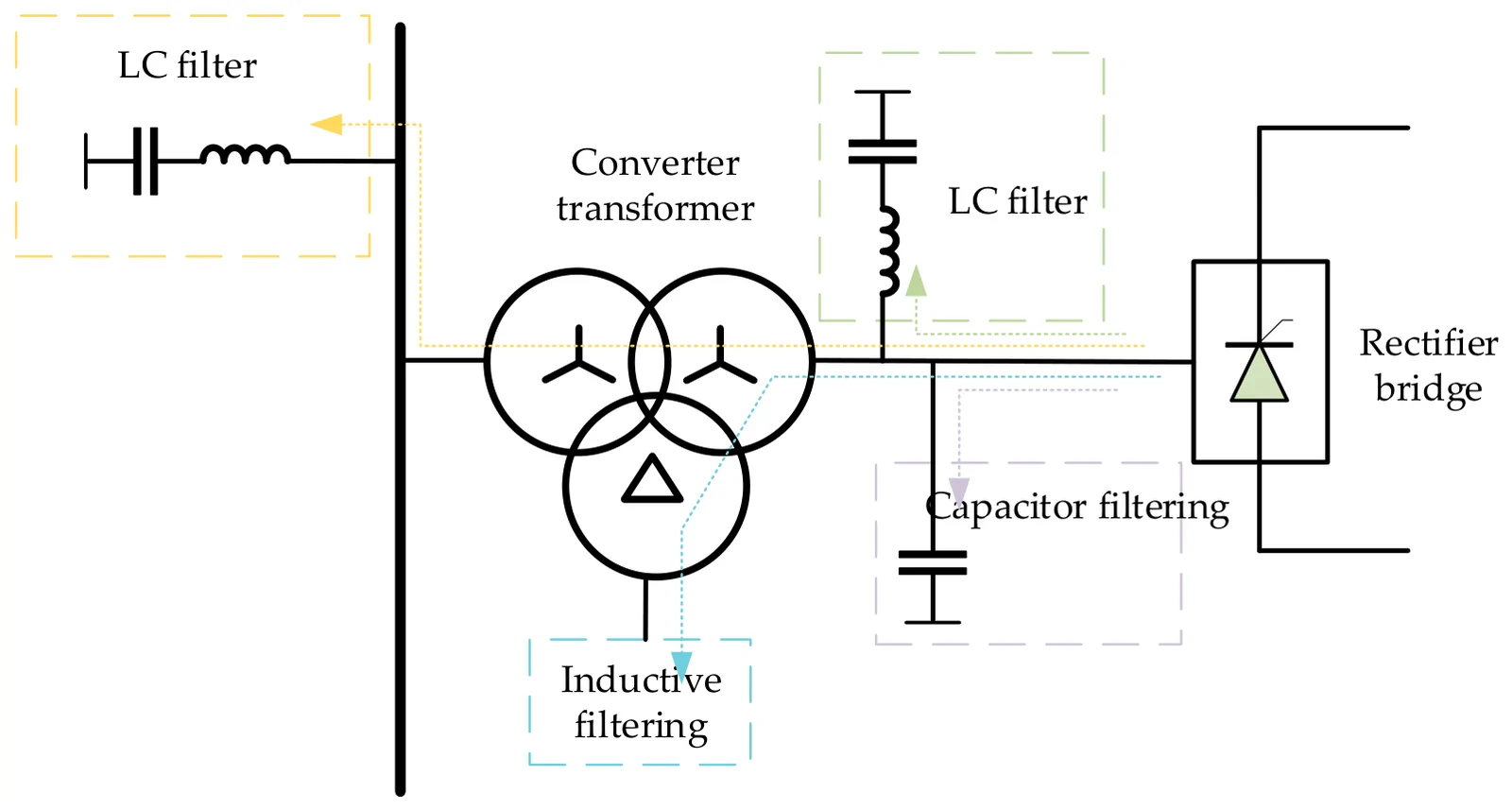
Harmonic Paths under Different Filtering Methods.

Based on the above model, a qualitative analysis is conducted on how the four filtering methods alter the spectrum *I_h_* of the source excitation *i*(*t*), thereby affecting the resulting vibration and noise. Figure 1 shows the circulation paths of harmonic currents under different filtering methods, where different colors are used to distinguish the corresponding filtering configurations.

The impedance of the LC filter (on the grid side) is *Z_LC_*(*jω*) = *jωL* + 1/(*jωC*).

For the harmonic current *I*_g,*h*_ flowing into the grid, its shunt ratio is determined by the relative magnitudes of the filter impedance and the grid impedance. However, the key point is that the valve-side current of the transformer *I*_valve,*h*_ is mainly determined by the converter modulation and is essentially unaffected by the downstream grid-side LC filter. Therefore, in the vibration and noise model, the excitation current spectrum *I_h_* remains almost unchanged, resulting in a very weak suppression effect.

The core of the induction filter is harmonic ampere-turn balance. Assuming the valve-side winding turns are *N_v_*, the filter winding turns are *N_c_*, and the impedance of the tuning branch approaches zero at the harmonic frequency *ω_h_*, then according to the magnetomotive force balance equation:

(16) $$I_{\mathrm{valve},h}N_{v}+I_{c,h}N_{c}+I_{m,h}N_{m}\approx 0$$

Among them, *I_m,h_* is the harmonic excitation current referred to the main winding, and *N_m_* is the number of turns of the main winding (usually the grid-side winding). Since the tuning branch provides a low-impedance path for the harmonic current, *I_c,h_* is large and its phase is nearly opposite to that of *I*_valve,*h*_, resulting in an extremely small resultant magnetomotive force *I_m_*_,*h*_*N_m_*. This means that the harmonic flux density amplitude *B_h_*∝∣*I_m,h_*∣ in the main core is significantly suppressed. Based on Equation (5), the excitation force components related to core magnetostriction (especially those containing *B_h_*) will be markedly reduced. However, the impact on the winding current *I*_valve,*h*_ itself is limited, so the suppression effect on the winding Lorentz force is relatively poor.

The capacitor filter is connected on the valve side between the converter and the transformer, and its equivalent impedance is *Z*_c_(*jω*) = 1/(*jωC*), with its magnitude decreasing as the frequency increases: ∣*Z_c_*∣ = 1/(*ωC*). This frequency-dependent characteristic determines that the capacitor filter has a stronger attenuation capability for high-frequency harmonics.

For the harmonic current *I*_inv,*h*_ output by the converter, the capacitor branch and the transformer branch form a parallel shunt relationship. The harmonic current *I*_valve,*h*_ flowing into the transformer satisfies:

(17) $$I_{\mathrm{valve},h}=I_{\mathrm{inv},h}\cdot \frac{Z_{c}(j\omega h)}{Z_{c}(j\omega h)+Z_{T}(j\omega h)}$$

where *Z_T_*(*jωh*) is the equivalent impedance of the transformer at the harmonic frequency (mainly leakage reactance, *Z_T_* ≈ *jωhL_σ_*) [29]. Since *Z_C_* decreases as the frequency increases, while *Z_T_* increases as the frequency increases, the shunt ratio ∣*I*_valve,*h*_/*I*_inv,*h*_∣ becomes significantly less than 1 for high-frequency harmonics.

Specifically, the higher the harmonic frequency *ωhs*, the smaller the capacitor impedance 1/(*ωhC*), and the stronger the low-impedance path effect provided for the harmonic current, resulting in a more significant attenuation of the amplitude of the harmonic current flowing into the transformer. Let the attenuation coefficient be:

(18) $$\alpha_{h}=\left|\frac{Z_{c}(j\omega h)}{Z_{c}(j\omega h)+Z_{T}(j\omega h)}\right|=\frac{1}{\sqrt{1+{\left(\omega^{2}h^{2}L_{\sigma }C\right)}^{2}}}$$

Therefore, capacitive filtering exhibits a frequency-selective attenuation characteristic for the harmonic current flowing into the transformer: for low-order harmonics (e.g., 5th and 7th), the harmonic angular frequency values are relatively small, resulting in limited attenuation; for medium- and high-frequency harmonics (e.g., 11th, 13th, 23rd, 25th and above), the harmonic angular frequency values are larger, leading to significant attenuation. Based on Equations (5) and (10), both the core magnetostrictive force and the winding Lorentz force are attenuated synchronously with the harmonic current. However, due to the frequency dependence of the attenuation coefficient, the excitation force components corresponding to medium- and high-frequency harmonics are attenuated more significantly.

Through detailed derivation starting from the coupled physical essence of electromagnetism, mechanics, and acoustics, this work clearly reveals the mathematical mechanism by which harmonics complicate the vibration and noise spectrum, and qualitatively demonstrates the different links in the coupling chain at which various filtering schemes act, as well as the differences in their effects. The analysis clarifies how changes in harmonic current components affect electromagnetic excitation, propagate through the vibration response, and ultimately influence acoustic radiation, thereby providing an interpretation of the suppression mechanisms and laying a solid theoretical foundation for experimental design and result analysis.

### 2.3. Simulation Calculation and Distribution Characteristics of Converter Transformer Vibration Under Harmonic Conditions

The rated flux density of the laboratory prototype converter transformer is 1.506 T. The main winding parameters, obtained from the design specifications of the 20 kVA laboratory prototype converter transformer used in this study, are listed in Table 1.

**Figure 2:**
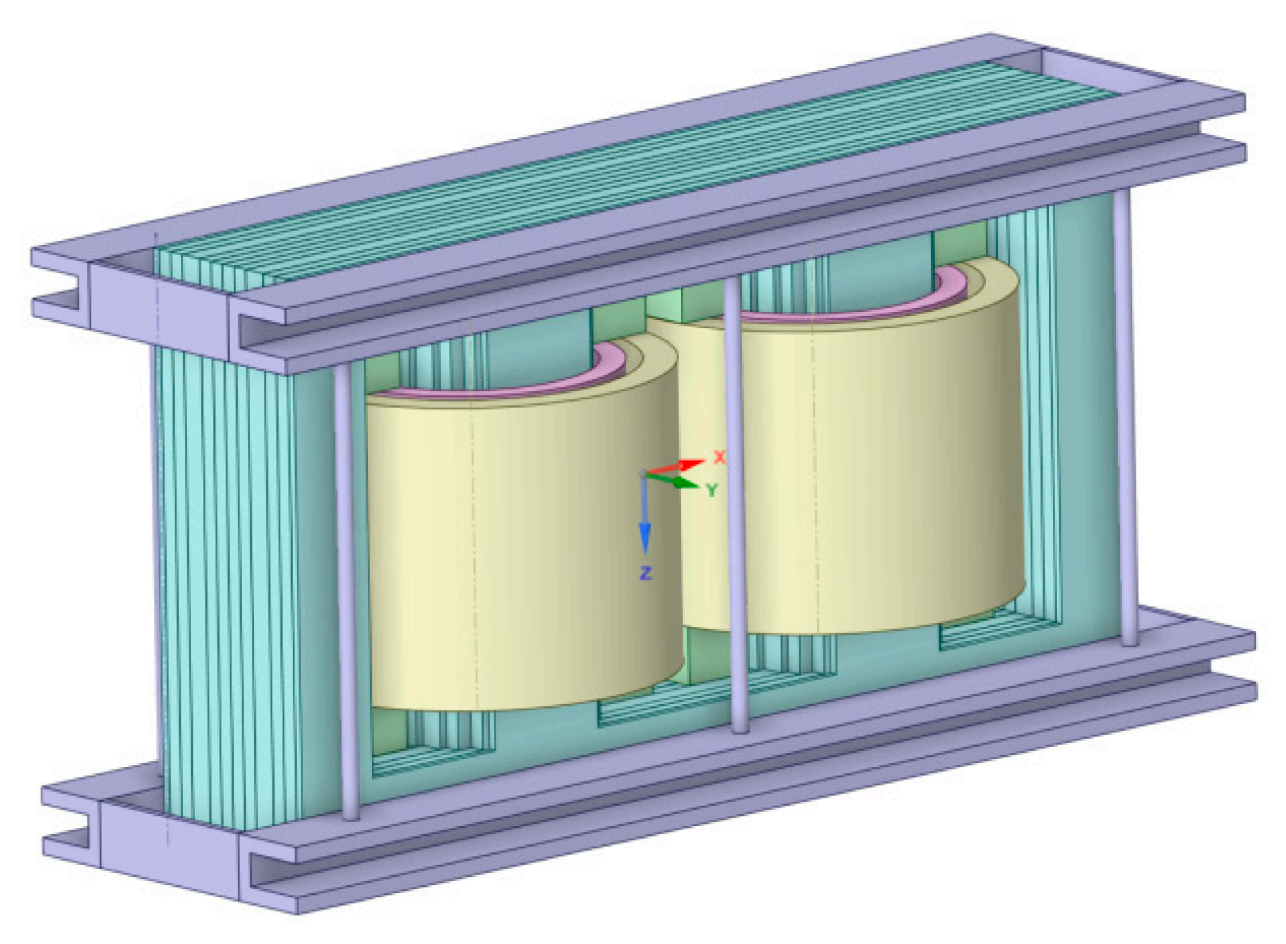
Three-dimensional finite-element structural model of the converter-transformer prototype.

Based on the structural configuration and design parameters of the converter-transformer prototype, a three-dimensional finite-element structural model was established for the subsequent multiphysical field analysis, as shown in Figure 2. This model represents the physical structure of the converter transformer used in the electromagnetic and structural simulations. Laminated silicon-steel cores exhibit direction-dependent mechanical properties. In this study, an equivalent isotropic model is adopted as an engineering simplification for comparative analysis under identical modeling conditions.

In the numerical analysis of the electromagnetic–structural fields of the transformer, different material parameters are required for the electromagnetic field and the structural mechanical field. The mechanical performance parameters of the converter transformer are listed in Table 2.

**Figure 3:**
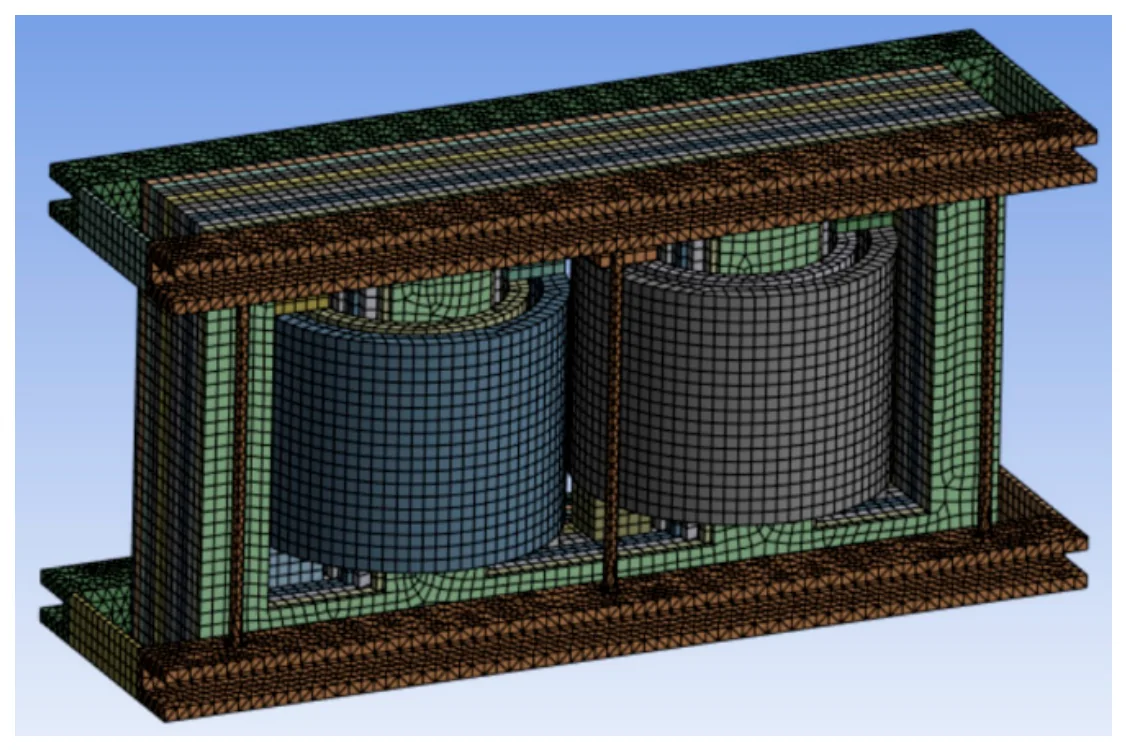
Mesh division of the converter transformer principle prototype.

In finite element simulations of oil-immersed transformers, proper mesh generation is essential for balancing computational efficiency and accuracy. In the proposed multi-physics simulation model, the laminated core has a complex geometry and requires mesh refinement to ensure accuracy. Meanwhile, the high- and low-voltage windings, structural steel, and spacers have simpler geometries, allowing for adaptive meshing to balance cost and accuracy. Figure 2 and Figure 3 show the structural model and mesh division of the converter-transformer prototype, respectively. In both figures, different colors are used to distinguish components with different material properties.

The excitation source uses measured harmonic data. The amplitudes and phases of individual harmonics are synthesized into a time-domain current waveform and applied to the windings to faithfully represent the complex current spectrum resulting from converter valve switching. Neumann boundary conditions are imposed on the outer surface of the solution domain, where the magnetic flux lines are set parallel to the boundary, simulating natural magnetic field decay in the far field. This approach prevents artificial disturbance to the internal field distribution while maintaining computational stability.

**Figure 4:**
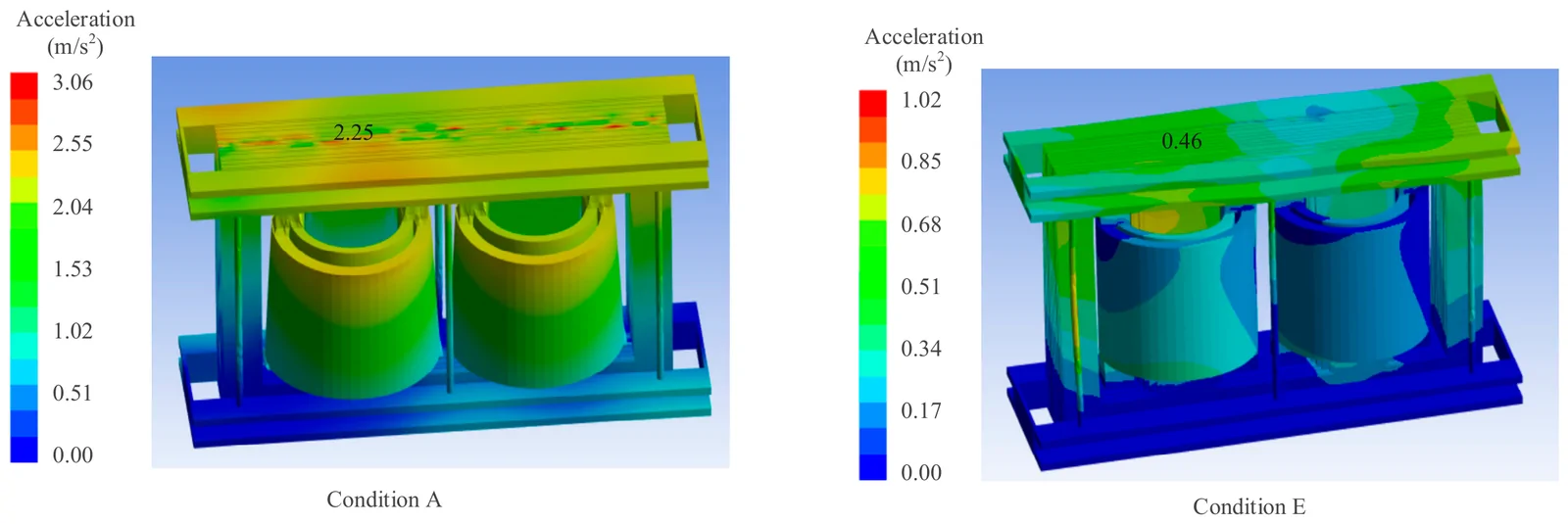
Vibration acceleration cloud diagrams of Condition A and Condition E.

Figure 4 shows the vibration acceleration cloud diagrams of the converter transformer principle prototype under the conditions without a filter (Condition A) and with a capacitive filter (Condition E).

As shown in Figure 4, the maximum vibration acceleration of the converter transformer is 3.06 m/s^2^ when no filter is applied, and 1.02 m/s^2^ when capacitive filtering is applied. This reduction indicates that capacitive filtering weakens harmonic electromagnetic excitation transmitted to the transformer structure, thereby reducing its vibration response. The vibration acceleration of the converter transformer is effectively suppressed, which is consistent with the theoretical analysis.

## 3. Experimental Verification

To verify the aforementioned theoretical analysis, a 12-pulse HVDC principle prototype experimental platform is designed and constructed. The developed test platform mainly consists of three subsystems: the DC transmission system, the transformer vibration and noise detection unit, and the backend centralized control unit. The platform also enables consistent switching among different filtering configurations, ensuring comparable operating conditions for subsequent vibration and noise measurements. This experimental system can be used to analyze the vibration response and acoustic characteristics of the converter transformer under harmonic excitation.

### 3.1. DC Transmission System

**Figure 5:**
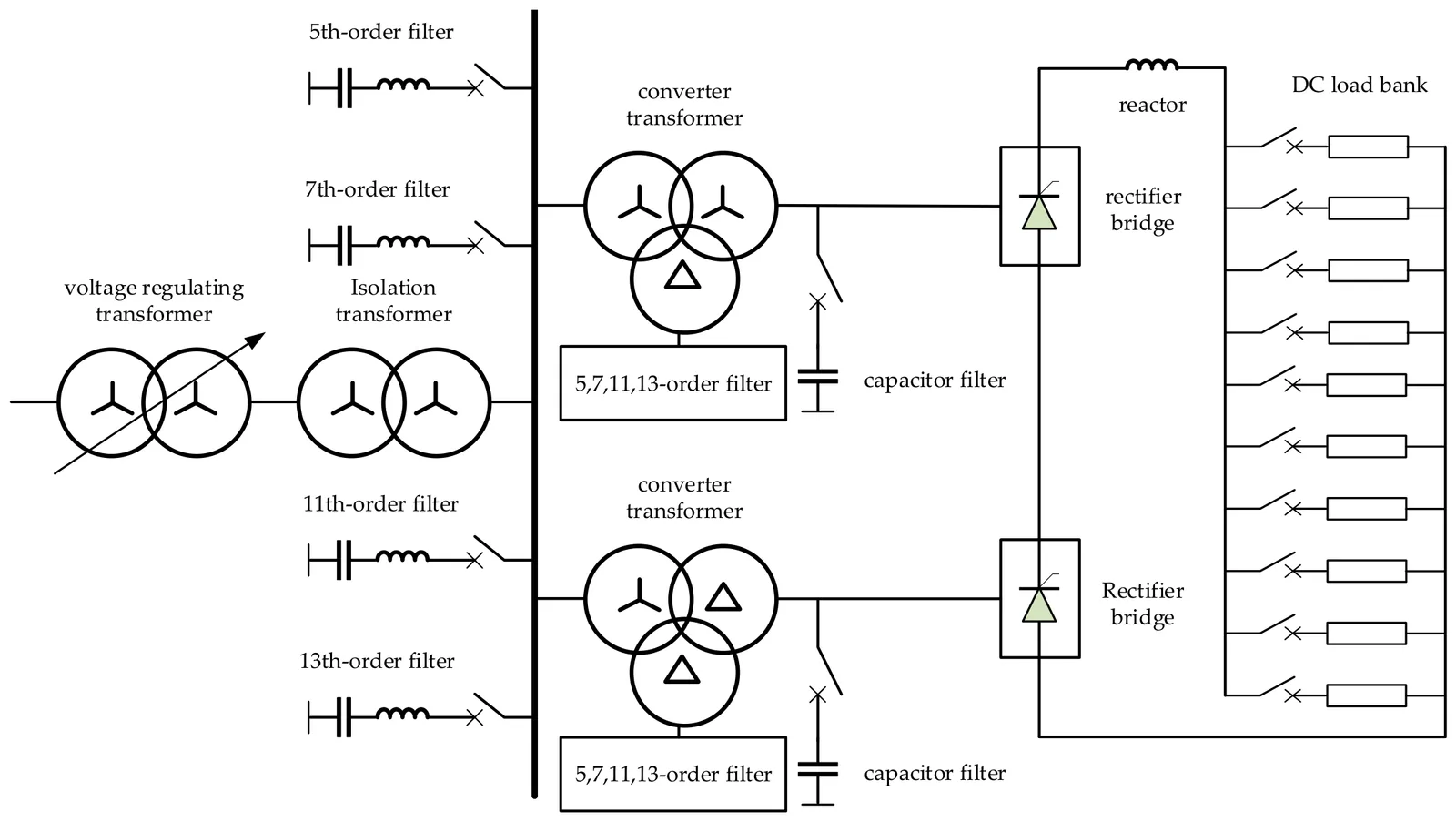
Engineering wiring diagram of the experimental platform.

As a key component of the test platform, the DC transmission unit integrates various types of high-voltage electrical equipment, specifically including a 10 kV voltage regulating and isolation transformer, system switchgear, converter transformer, shunt capacitor modules, current limiting reactor devices, a twelve-pulse rectifier module, a smoothing reactor, and a DC load. The engineering wiring diagram of the experimental platform is shown in Figure 5. During operation, the regulating and isolation transformer provides adjustable input voltage and electrical isolation, while the smoothing reactor suppresses DC-side current ripple and stabilizes the transmission current. The modular SSconfiguration of the filtering branches allows the grid-side LC filter, valve-side LC filter, inductive filter, and valve-side capacitive filter to be independently connected according to the required experimental operating condition.

The converter transformer is of the single-phase four-limb type, featuring both Y-connected and Δ-connected winding configurations, with three transformers provided for each configuration. All transformers are designed as two-winding transformers. A 12-pulse rectifier system is achieved through voltage-phase shifting of the load windings.

The LC filters are 5th, 7th, 11th, and 13th single-tuned passive filters, and the capacitance in the capacitor filter is 71 μF.

The physical arrangement and sensor positions of the two transformer units were maintained unchanged throughout all operating conditions to ensure consistent comparative measurements.

### 3.2. Vibration and Noise Test Platform

**Figure 6:**
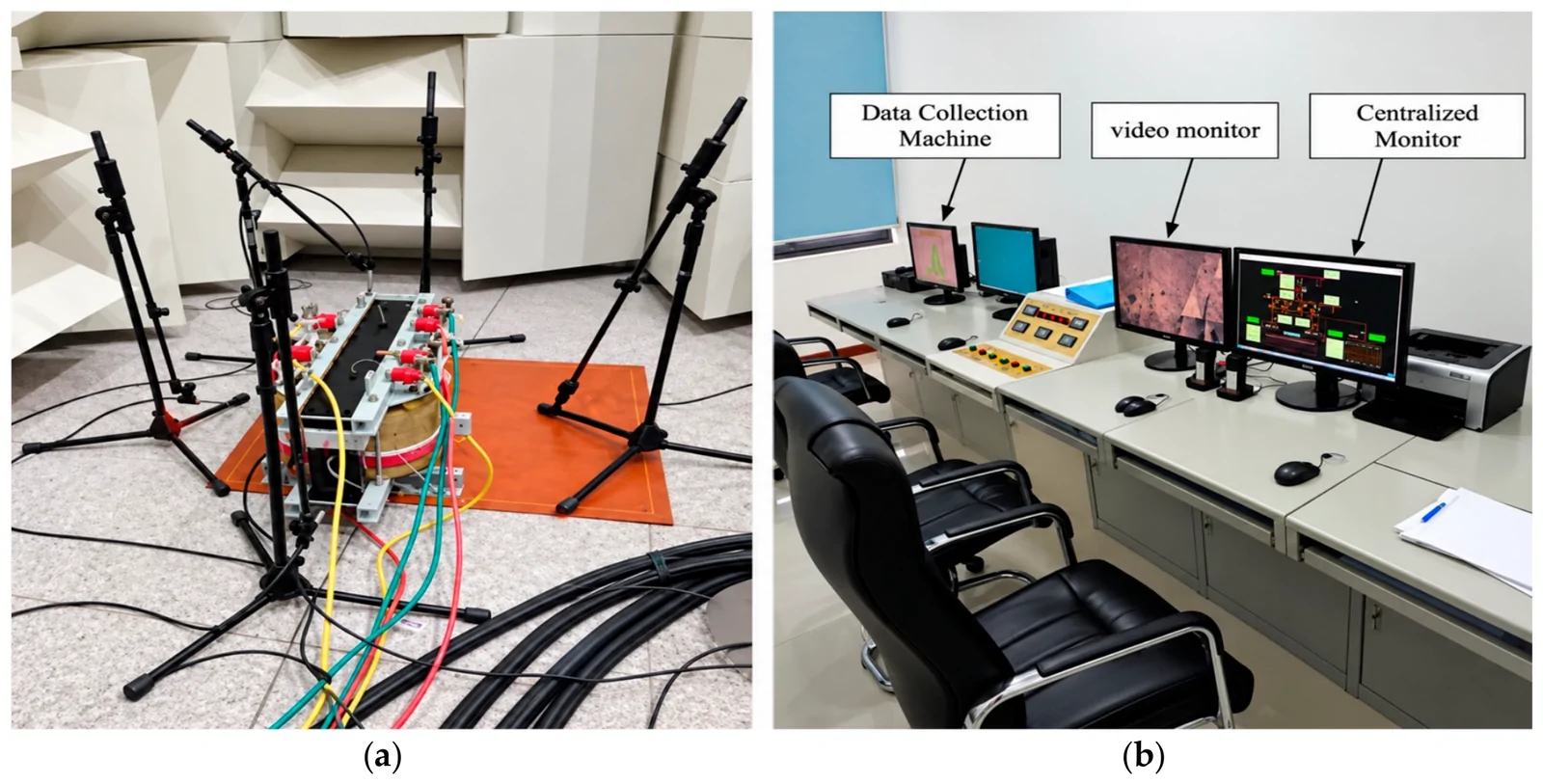
Vibration and noise test system of the converter transformer and the central integrated control system. (**a**) Vibration and noise testing system for converter transformer. (**b**) Centralized control system.

The converter transformer vibration and noise detection device consists of a semi-anechoic chamber and a multi-channel acquisition system, with the overall layout shown in Figure 6a. The experimental system comprises six single-phase converter transformers, while the vibration and noise measurements are conducted on one representative transformer.

The semi-anechoic chamber provides a test environment approximating a free sound field, where the influence of sound reflections inside the chamber is negligible, and the background noise is below 17.9 dB. It can effectively shield external interference and significantly improve the accuracy of transformer vibration and acoustic measurements. The detection system is equipped with high-performance sound source localization equipment from Brüel & Kjær (B&K) of Virum, Denmark, which can be expanded to 30 synchronous acquisition channels. The vibration acceleration was measured using B&K Type 4534-B-001 uniaxial CCLD accelerometers(Hottinger Brüel & Kjær A/S, Virum, Denmark), with a measurement range of ±71 g and a frequency range of 0.2 Hz–12.8 kHz. The vibration signals were acquired using B&K LAN-XI Type 3050-A-060 six-channel input modules (Hottinger Brüel & Kjær A/S, Virum, Denmark) installed in a Type 3660-C-100 front-end frame(Hottinger Brüel & Kjær A/S, Virum, Denmark). The acquisition modules provide a sampling rate of 131 ksamples/s and a frequency range of DC 51.2 kHz, enabling synchronous multi-channel vibration measurements.

The central integrated control platform integrates multiple modules, including DC monitoring, vibration and noise detection, primary equipment control, and video surveillance. It has functions such as remote voltage regulation, circuit breaker control, DC system adjustment, remote operation of filtering devices, real-time data collection, signal tracking, and test site image monitoring, achieving centralized intelligent management of the entire test process. This system has a high degree of integration, effectively enhancing the overall test efficiency and safety level of the platform. Figure 6b shows the central integrated control system.

**Figure 7:**
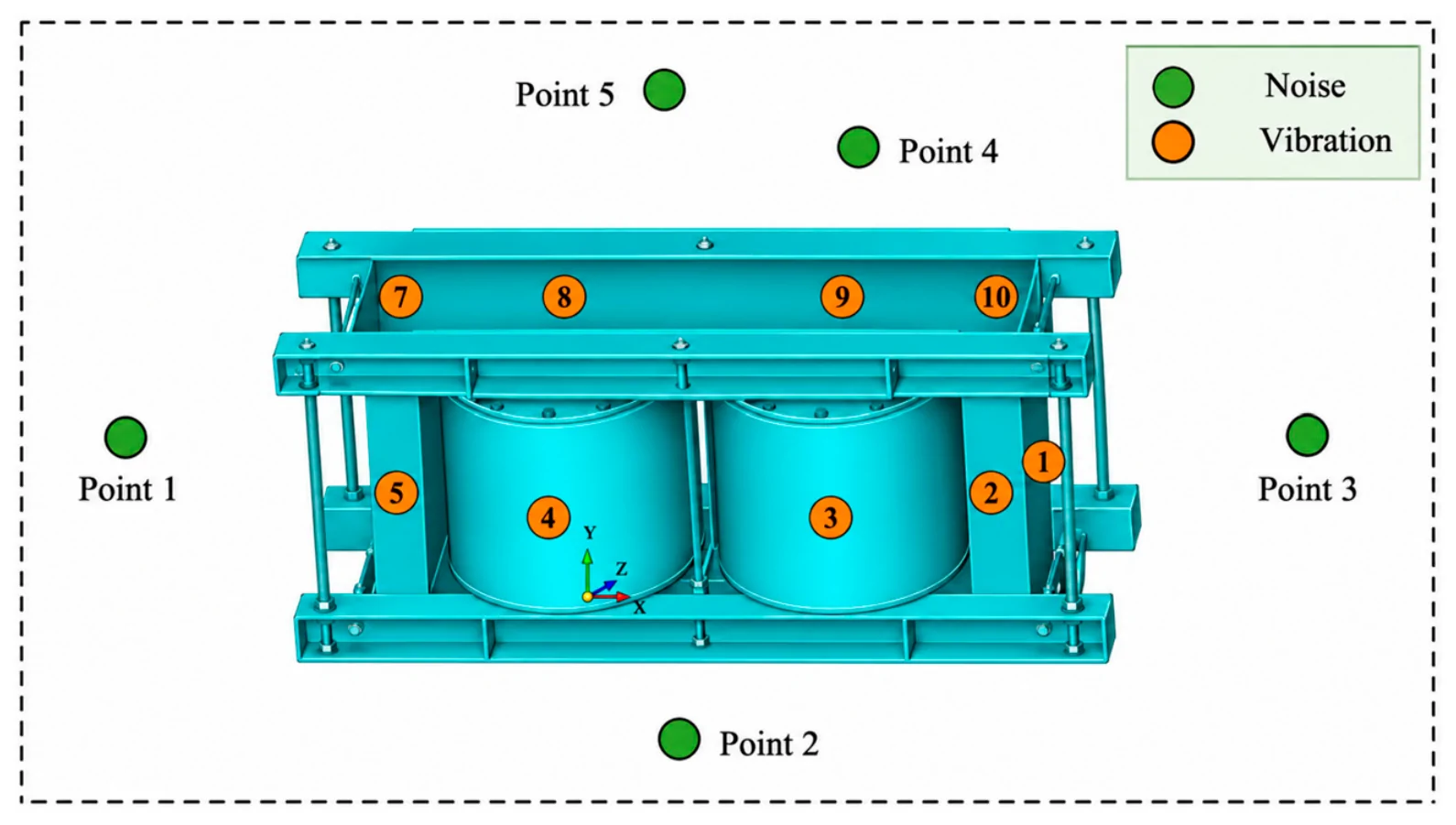
Installation points of sound and vibration sensors.

Figure 7 illustrates the mounting locations of the five acoustic sensors and ten vibration sensors. In Figure 7, the green markers labeled Points 1 to 5 indicate the acoustic measurement locations, while the orange markers numbered 1to 10 indicate the vibration measurement locations. (Due to line-of-sight limitations, the wide face of the side yoke and the position symmetric to ① are not indicated in the figure). The vibration measurement points were selected to cover the main vibration-source and vibration-transmission regions of the converter transformer, including representative positions associated with the core, windings, clamps, and tank wall. The acoustic measurement points were arranged according to GB/T 1094.10-2022 [30] at a distance of 30 cm from the reference emission surface. Recent converter-transformer measurements also show that the location of vibration measurement points can noticeably affect the observed spectral characteristics [31].

## 4. Experimental Results and Analysis of Converter Transformer Vibration and Noise Under Different Filtering Methods

Based on the established 12-pulse HVDC principle prototype experimental platform, at the State Grid Electric Power Research Institute, China, the test data under five experimental operating conditions are systematically presented and deeply analyzed. The analysis follows a progressive logic from electrical quantities to mechanical quantities to acoustic quantities, aiming to reveal the suppression mechanisms and effect differences of different filtering methods on converter transformer vibration and noise, and to examine the relationship among harmonic suppression, vibration attenuation, and noise reduction.

### 4.1. Experimental Scheme Design and Filtering Effects Under Different Filtering Methods

To ensure comparability among the different filtering schemes, the DC-side voltage was used as the regulation reference. The regulating transformer was adjusted to maintain approximately the same DC voltage target under the five operating conditions.

Condition A (Benchmark condition): No filtering device is activated. The vibration and noise data of the converter transformer under pure 12-pulse harmonic excitation are recorded.

Condition B (LC grid-side filtering condition): The grid-side LC filter is activated. The DC load power is maintained at the same level as in Condition A.

Condition C (LC valve-side filtering condition): The valve-side LC filter branch is activated. The load power is also kept constant.

Condition D (Inductive filtering condition): The inductive filter is activated. The load power is kept constant.

Condition E (Valve-side capacitive filtering condition): The valve-side capacitor filter is activated. The load power is kept constant.

Under different filtering conditions, spectrum analysis is performed on the valve-side current of the converter transformer, with emphasis on the variation patterns of characteristic harmonics (11th, 13th, 23rd, 25th, etc.) and the differences in total harmonic distortion (THD). The valve-side current was experimentally measured under the five operating conditions. Table 3 presents the measured total harmonic distortion (THD) of the transformer valve-side current and the content ratios of the selected harmonic components under these operating conditions.

**Figure 8:**
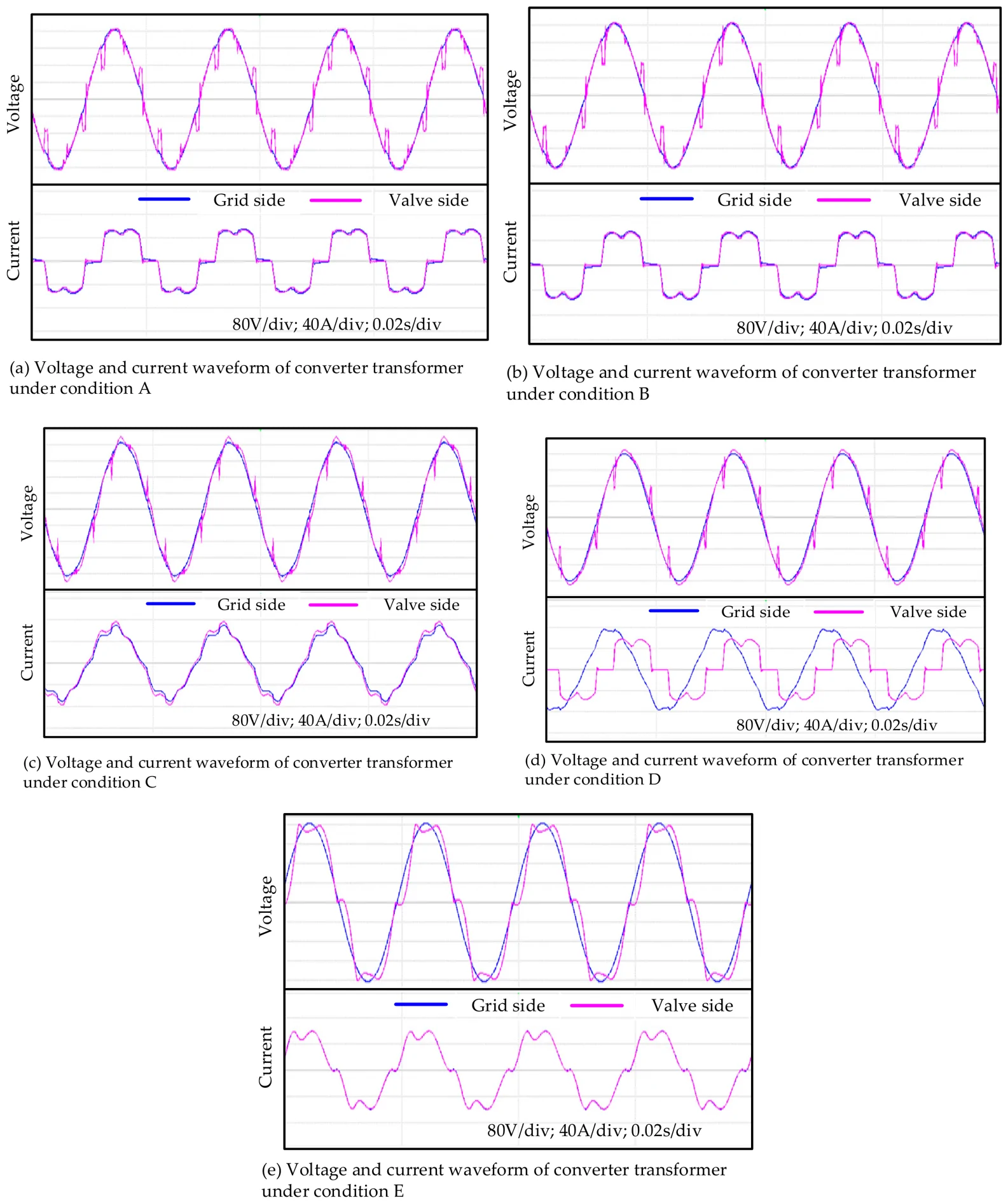
Voltage and current waveforms of the converter transformer under different operating conditions.

The voltage and current waveforms of the converter transformer under different operating conditions are shown in Figure 8.

The pulse-like distortions observed in the voltage and current waveforms in Figure 8 are mainly associated with the periodic commutation process of the 12-pulse converter. During commutation, the current is transferred between converter phases, causing transient deviations in the transformer-side voltage and current from ideal sinusoidal waveforms. The periodic repetition of this process produces the pulse-like waveform distortions and contributes to the characteristic harmonic components of the converter system. More generally, switching operations in power-electronic systems can also introduce high-frequency harmonic components. For the 12-pulse converter, these components mainly occur at harmonic orders of 12k ± 1, including the 11th, 13th, 23rd, and 25th harmonics considered in this study. The corresponding harmonic contents and THD values under different filtering conditions are further quantified in Table 3 and Table 4.

It should be noted that the DC-side voltage was used as the regulation reference to maintain comparable load conditions among the five cases. The differences in the grid-side and valve-side RMS quantities are mainly attributed to changes in the equivalent system impedance after the connection of different filtering branches.

The experimental results show that under the no-filter condition (Condition A), the current waveform exhibits significant distortion, with the spectrum dominated by the 11th and 13th harmonics, accompanied by certain amplitudes of higher-order harmonic components. At this time, the system THD is at a relatively high level, indicating that the harmonics generated during the commutation process are not effectively suppressed.

After introducing grid-side LC filtering (Condition B), a reduction in grid-side harmonic current can be observed, but the improvement in the valve-side current waveform is not significant. This indicates that the grid-side filter mainly acts at the grid interface and has a limited effect on the harmonic distribution inside the converter transformer. The reason is that the harmonics have already flowed through the transformer windings before entering the filter, thereby generating electromagnetic effects within the transformer.

In contrast, valve-side LC filtering (Condition C) acts directly near the harmonic source, resulting in a more significant improvement in the current waveform. Experimental data show that the THD decreases by approximately 60% compared with the no-filter condition, and the amplitudes of low-order characteristic harmonics are significantly reduced. However, it should be noted that this filtering method has a limited suppression effect on high-frequency harmonics, and a certain amplitude of high-order components still remains in the spectrum.

Under the inductive filtering condition (Condition D), the harmonic current exhibits a distinct frequency-selective attenuation characteristic. Specifically, certain specific frequencies (such as the 11th or 13th harmonics) are significantly weakened, while other frequency components show relatively small changes. This is because inductive filtering achieves “targeted suppression” of specific harmonics through a magnetomotive force cancellation mechanism.

Under the inductive filtering condition (Condition D), the valve-side current THD decreases only slightly, from 26.71% to 24.67%, and the individual harmonic components are not uniformly attenuated. This is consistent with the magnetomotive-force cancellation mechanism of inductive filtering, which primarily suppresses the resultant harmonic magnetomotive force rather than directly reducing all harmonic components of the valve-side current.

Under the valve-side capacitive filtering condition (Condition E), the high-order harmonic components are strongly attenuated. The overall valve-side current THD is 17.93%, which is higher than the 10.03% obtained under valve-side LC filtering (Condition C). Therefore, Condition E does not produce the lowest overall THD. Nevertheless, it provides effective attenuation of several medium- and high-order harmonic components. This observation indicates that vibration and noise suppression cannot be evaluated solely by total harmonic distortion. In addition to the overall THD, the distribution of individual harmonic components should also be considered. The capacitive filter provides a low-impedance path on the valve side, allowing part of the harmonic current to be shunted before entering the transformer and thereby reducing harmonic excitation at the source.

### 4.2. Experimental Measurement Results of Converter Transformer Vibration Under Different Filtering Methods

**Figure 9:**
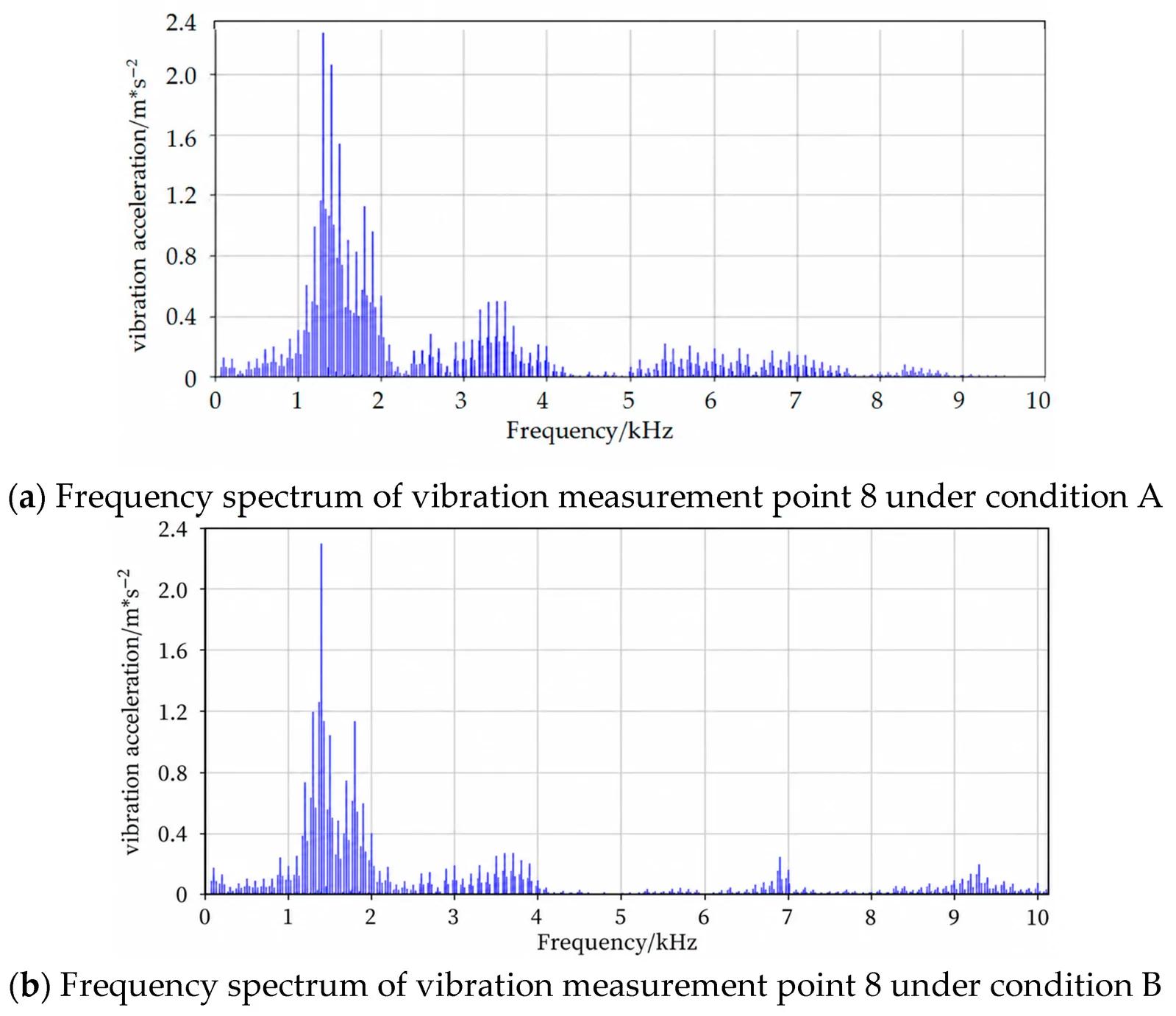

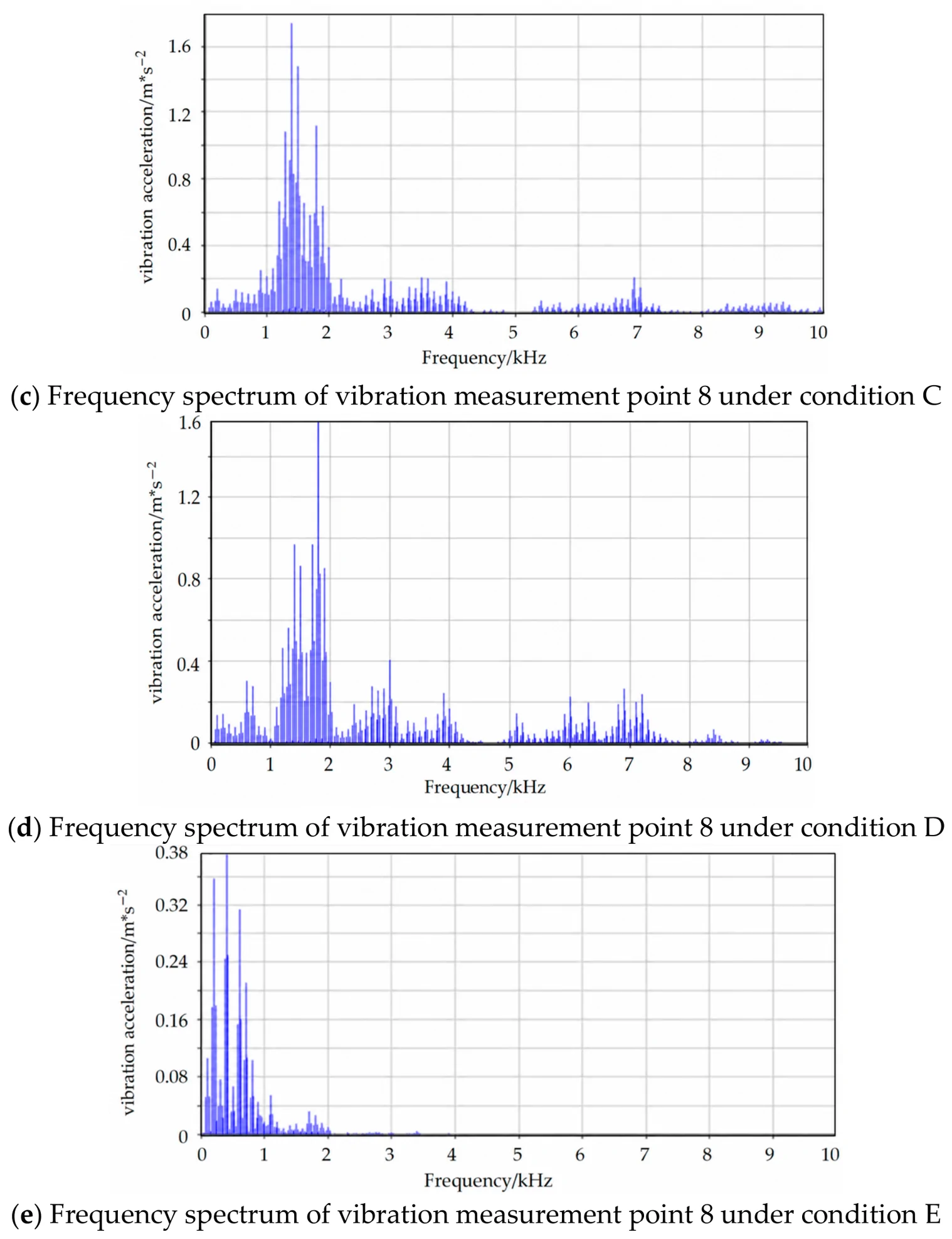
Spectra of vibration measurement point 8 under different operating conditions.

Based on the harmonic analysis, the vibration acceleration at key measurement points of the converter transformer (core, windings, and tank surface) is further tested to analyze the influence of different filtering methods on the vibration level. The selected vibration measurement points are 1, 3, 4, 6, 7, 8, and 9. Figure 9 shows the vibration acceleration spectrum at measurement point 8.

Table 5 lists the vibration acceleration data of the converter transformer under five operating conditions.

The following trends can be observed from Table 5:

Under the no-filter condition (Condition A), the vibration acceleration at each measurement point is generally at a relatively high level, with Point 1, Point 8, and Point 9 reaching 2.77 m/s^2^, 2.28 m/s^2^, and 2.38 m/s^2^, respectively, which are significantly higher than those at other measurement points. This indicates that these measurement locations exhibit relatively strong vibration responses under the no-filter condition.

After introducing grid-side LC filtering (Condition B), the vibration amplitudes at all measurement points decrease, but the overall reduction is limited. For example, Point 1 decreases from 2.77 m/s^2^ to 2.41 m/s^2^ (a reduction of approximately 13%), Point 8 decreases from 2.28 m/s^2^ to 2.07 m/s^2^ (approximately 9%), and Point 9 decreases from 2.38 m/s^2^ to 1.97 m/s^2^ (approximately 17%). This result indicates that although grid-side filtering attenuates the system harmonics to some extent, it has little effect on the internal electromagnetic excitation of the transformer, so the vibration improvement is not significant.

In contrast, valve-side LC filtering (Condition C) exhibits a more significant vibration suppression effect. The vibrations at multiple key measurement points are notably reduced, among which Point 6 decreases from 0.79 m/s^2^ to 0.45 m/s^2^ (a reduction of approximately 43%), Point 7 decreases from 1.79 m/s^2^ to 1.47 m/s^2^ (approximately 18%), and Point 8 decreases from 2.28 m/s^2^ to 1.83 m/s^2^ (approximately 20%). This indicates that effective suppression of low-order harmonics significantly weakens the main electromagnetic excitation sources, resulting in a notable vibration reduction at some measurement points.

Under the inductive filtering condition (Condition D), the vibration changes exhibit a certain degree of non-uniformity. Some measurement points, such as Point 9 (1.92 m/s^2^), are close to those in Condition C, while Point 7 (1.77 m/s^2^) is close to the no-filter level. This indicates that the influence of inductive filtering on vibration has a distinct frequency-selective characteristic, suppressing only the vibration modes corresponding to certain harmonic excitations, while the overall vibration energy reduction is limited.

Under the valve-side capacitive filtering condition (Condition E), the vibration suppression effect is the most significant. The vibrations at all key measurement points decrease substantially, among which Point 1 decreases from 2.77 m/s^2^ to 0.82 m/s^2^ (a reduction of approximately 70%), Point 7 decreases from 1.79 m/s^2^ to 0.43 m/s^2^ (approximately 76%), Point 8 decreases from 2.28 m/s^2^ to 0.42 m/s^2^ (approximately 82%), and Point 9 decreases from 2.38 m/s^2^ to 0.45 m/s^2^ (approximately 81%). Meanwhile, the vibration levels at each measurement point tend to be consistent, indicating a more uniform vibration-response distribution.

The simulated vibration acceleration values at measurement Point 8 are extracted and compared with the measured data. Table 6 presents the comparison results between simulation and measurement under the five operating conditions. This comparison is intended primarily to validate the relative vibration-response trends among the different filtering conditions rather than to provide a complete spatial validation of the finite-element model.

The error between the simulation and measurement results is less than 10%, which verifies the correctness and effectiveness of the established finite element model and analysis approach, indicating that both the vibration test and simulation data adopted in this paper have high engineering credibility.

Comprehensive analysis shows that the variation in vibration response is not only related to the magnitude of harmonics but also closely related to the harmonic spectrum structure. Although valve-side LC filtering (Condition C) achieves a lower THD, capacitive filtering (Condition E) provides substantially greater vibration suppression. This result indicates that the vibration response depends not only on the overall THD but also on the distribution of individual harmonic components.

### 4.3. Experimental Measurement Results of Converter Transformer Noise Under Different Filtering Methods

Noise is the final manifestation of vibration energy radiated into the air and is the ultimate indicator of engineering concern. By analyzing the sound pressure level around the transformer, the noise reduction effect of different filtering schemes can be evaluated.

According to the GB/T 1094.10 standard, five measurement points are arranged at a distance of 30 cm from the reference emission surface of the transformer (the specific arrangement positions are shown in Figure 7). The A-weighted sound pressure levels under each operating condition are measured, and their average value is taken as a comprehensive indicator characterizing the noise level. The results are listed in Table 7.

**Figure 10:**
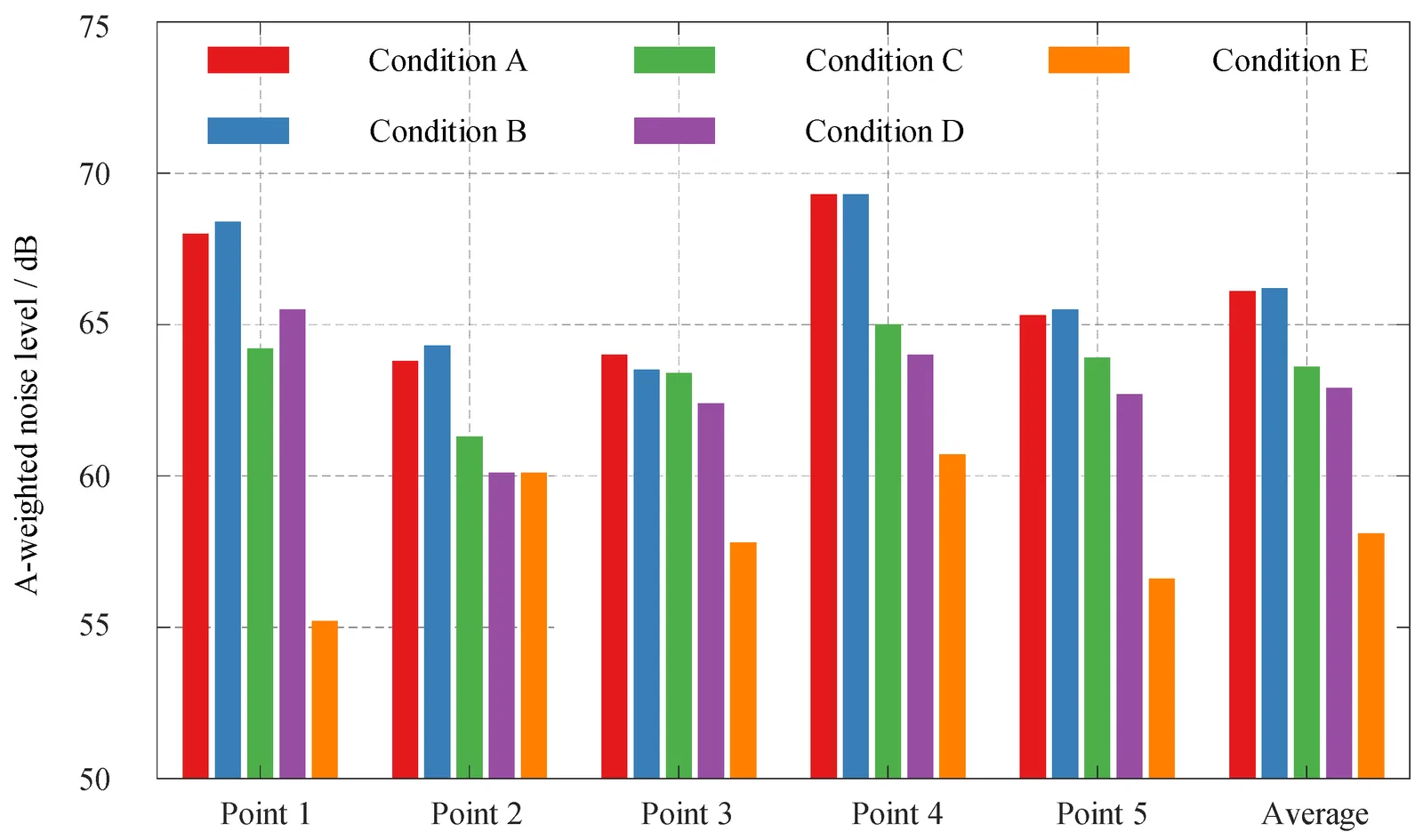
Noise values and average values at various points.

For a more intuitive comparison of the noise data under different operating conditions, the data in Table 7 are converted into Figure 10.

From Table 7 and Figure 10, the following can be observed:

Under the no-filter condition (Condition A), the average sound pressure level of the converter transformer is 66.1 dB, with differences in noise levels among the measurement points. Point 1 and Point 4 reach 68.0 dB and 69.3 dB, respectively. After introducing grid-side LC filtering (Condition B), the average sound pressure level changes only slightly to 66.2 dB, indicating that although this method can reduce the grid-side harmonic current, the actual noise suppression effect is extremely limited because the harmonics have already generated electromagnetic forces and vibrations inside the transformer before entering the filter.

Valve-side LC filtering (Condition C) reduces the average sound pressure level to 63.6 dB, with Point 4 and Point 1 decreasing by 4.3 dB and 2.5 dB, respectively, indicating that the attenuation of low-order characteristic harmonics (e.g., 11th and 13th) can effectively reduce electromagnetic excitation and vibration sound radiation, although high-frequency harmonics still contribute to local noise.

Inductive filtering (Condition D) further reduces the average sound pressure level to 62.9 dB, with some measurement points such as Point 2 decreasing by 3.7 dB, while Point 1 remains at 65.5 dB, reflecting the spatial non-uniformity of the noise reduction effect.

Valve-side capacitive filtering (Condition E) exhibits the most significant effect, reducing the average sound pressure level to 58.1 dB, with Point 1 and Point 4 decreasing by 12.8 dB and 8.6 dB, respectively, and the noise levels at each measurement point tend to be uniform. This method not only effectively reduces the amplitude of harmonic currents but also optimizes the harmonic spectrum structure, weakening the electromagnetic excitation at the source and avoiding the coupling between harmonic frequencies and structural natural frequencies, thereby significantly suppressing vibration response and sound radiation intensity. The sound-pressure-level data are primarily used for comparative evaluation among the five operating conditions under consistent measurement conditions; repeated-test statistics and confidence intervals were not included in the present study.

Based on the above comprehensive analysis, it can be seen that the variation trend of noise level is highly consistent with the vibration response, which further validates the physical transmission process from harmonic excitation to electromagnetic force, structural vibration, and finally sound radiation. Among the different approaches, simply reducing the harmonic amplitude, such as in grid-side filtering, has a limited effect on noise improvement. In contrast, suppressing harmonics at the source and optimizing the spectral distribution, as achieved by valve-side capacitive filtering, provides the most effective noise reduction. Therefore, in engineering applications, priority should be given to filtering schemes that can simultaneously reduce harmonic amplitude and improve spectral characteristics, so as to achieve coordinated control of vibration and noise.

## 5. Conclusions

Based on a 12-pulse HVDC experimental platform, this paper establishes an analytical framework following the physical process of harmonic excitation, electromagnetic force generation, structural vibration, and acoustic radiation. The vibration and noise characteristics of the converter transformer under different filtering methods are then systematically investigated. The main conclusions are as follows:

(1) The filter location has a decisive influence on the noise reduction effect. Configuring a filter only on the grid side is insufficient to reduce the vibration and noise of the transformer itself, whereas valve-side filtering can attenuate harmonic currents at the source, which is the key to achieving effective noise reduction.
(2) The mechanisms of different filtering methods differ significantly. Inductive filtering exhibits frequency-selective suppression characteristics, while valve-side capacitive filtering achieves source-level attenuation through harmonic current shunting, offering greater advantages in electromagnetic excitation suppression.
(3) Based on the comprehensive experimental results, the noise reduction capability of each scheme is ranked as follows: the valve-side capacitive filtering scheme shows the strongest noise-reduction capability, followed by inductive filtering, valve-side LC filtering, and grid-side LC filtering. The valve-side capacitive filtering scheme achieves the best noise reduction effect, with a reduction in the average A-weighted sound pressure level of approximately 8.0 dB.

The experiments in this paper are conducted on a 12-pulse principle prototype and do not account for factors such as temperature rise, load fluctuations, and aging in practical engineering. Future work can carry out field tests at higher voltage levels and over longer time scales, and explore the synergistic noise reduction mechanisms of active filtering and hybrid filtering.

## Figures and Tables

**Table 1:** Parameters of the Four-Limb Converter Transformer Principle Prototype.

Parameter	Grid-Side Winding	Valve-Side Winding	Filter Winding
Rated capacity (kVA)	20	20	10
Rated voltage (V)	220	220	380
Rated current (A)	91.16	91.16	26.32
Short-circuit impedance (%)	9	9	0.04

**Table 2:** The mechanical performance parameters.

Material Name	Density (kg/m^3^)	Elastic Modulus (GPa)	Poisson’s Ratio
Silicon steel sheet	7290	EX = EY = 207, EZ = 166	0.25
Structural steel	7850	206	0.3

**Table 3:** Comparison of transformer valve-side current THD and selected harmonic content ratios under five operating conditions.

Harmonic Order	Condition A	Condition B	Condition C	Condition D	Condition E
RMS value	39.06	38.20	42.08	40.87	40.96
3	0.07	0.06	0.69	0.15	0.23
5	9.07	9.22	2.21	8.25	6.94
7	3.50	3.29	3.45	2.60	2.32
11	2.80	2.96	0.33	2.54	0.47
13	1.81	1.82	0.09	2.13	0.19
17	1.25	1.45	0.26	2.02	0.11
19	0.93	1.09	0.20	1.46	0.05
23	0.53	0.73	0.24	1.32	0.05
25	0.39	0.64	0.21	1.07	0.03
29	0.24	0.32	0.20	0.91	0.02
31	0.10	0.32	0.19	0.80	0.02
35	0.28	0.11	0.18	0.64	0.01
37	0.06	0.11	0.17	0.59	0.01
41	0.29	0.17	0.16	0.44	0.01
43	0.14	0.08	0.15	0.43	0.01
47	0.23	0.24	0.15	0.29	0.00
49	0.18	0.16	0.13	0.30	0.00
THD	26.71	27.75	10.03	24.67	17.93

**Table 4:** Root mean square (RMS) values and THD of grid-side and valve-side voltages and currents of the converter transformer under five operating conditions.

Condition	Grid-Side Voltage		Grid-Side Current		Valve-Side Voltage		Valve-Side Current	
	Voltage (V)	THD	Current (A)	THD	Voltage (V)	THD	Current (A)	THD
Condition A	230.49	2.65	39.86	23.85	221.21	16.89	39.06	26.71
Condition B	230.12	1.89	39.17	24.86	220.30	16.83	38.20	27.75
Condition C	231.70	1.31	38.89	9.93	237.76	7.01	42.08	10.03
Condition D	226.61	1.10	40.37	6.27	232.18	10.89	40.87	24.67
Condition E	231.86	1.17	41.07	17.87	234.62	13.75	40.96	17.93

**Table 5:** Vibration acceleration of the converter transformer under five operating conditions (m/s^2^).

Condition	Point 1	Point 3	Point 4	Point 6	Point 7	Point 8	Point 9
Condition A	2.77	0.29	0.29	0.79	1.79	2.28	2.38
Condition B	2.41	0.20	0.25	0.75	1.74	2.07	1.97
Condition C	2.26	0.20	0.18	0.45	1.47	1.83	1.96
Condition D	2.25	0.25	0.22	0.55	1.77	1.61	1.92
Condition E	0.82	0.21	0.23	0.49	0.43	0.42	0.45

**Table 6:** Comparisons of simulated and measured vibration acceleration at measurement point 8 (m/s^2^).

Condition	Simulation	Measurement	Error (%)
Condition A	2.25	2.28	1.3
Condition B	1.89	2.07	8.7
Condition C	1.66	1.83	9.3
Condition D	1.73	1.61	7.5
Condition E	0.46	0.42	9.5

**Table 7:** Comparison of transformer A-weighted sound pressure levels under five operating conditions (dB).

Condition	Point 1	Point 2	Point 3	Point 4	Point 5	Average
Condition A	68.0	63.8	64.0	69.3	65.3	66.1
Condition B	68.4	64.3	63.5	69.3	65.5	66.2
Condition C	64.2	61.3	63.4	65.0	63.9	63.6
Condition D	65.5	60.1	62.4	64.0	62.7	62.9
Condition E	55.2	60.1	57.8	60.7	56.6	58.1

## Data Availability

Dates are available from the authors upon request.
